# Molecular Links between Flowering and Abiotic Stress Response: A Focus on *Poaceae*

**DOI:** 10.3390/plants12020331

**Published:** 2023-01-10

**Authors:** Daniele Chirivì, Camilla Betti

**Affiliations:** Department of Biosciences, University of Milan, Via Celoria 26, 20133 Milan, Italy

**Keywords:** abiotic stress, climate change, cereal crops

## Abstract

Extreme temperatures, drought, salinity and soil pollution are the most common types of abiotic stresses crops can encounter in fields; these variations represent a general warning to plant productivity and survival, being more harmful when in combination. Plant response to such conditions involves the activation of several molecular mechanisms, starting from perception to signaling, transcriptional reprogramming and protein modifications. This can influence the plant’s life cycle and development to different extents. Flowering developmental transition is very sensitive to environmental stresses, being critical to reproduction and to agricultural profitability for crops. The *Poacee* family contains some of the most widespread domesticated plants, such as wheat, barley and rice, which are commonly referred to as cereals and represent a primary food source. In cultivated *Poaceae*, stress-induced modifications of flowering time and development cause important yield losses by directly affecting seed production. At the molecular level, this reflects important changes in gene expression and protein activity. Here, we present a comprehensive overview on the latest research investigating the molecular pathways linking flowering control to osmotic and temperature extreme conditions in agronomically relevant monocotyledons. This aims to provide hints for biotechnological strategies that can ensure agricultural stability in ever-changing climatic conditions.

## 1. Introduction

Due to their sessile nature, plants cannot move away from unfavorable circumstances they encounter throughout their life cycle. For this reason, they must ensure immediate and coordinated stress response by modulating the expression and activity of several molecular players, from membrane receptors to transcription factors [1,2,3,4]. In fact, the plant response to abiotic stress is a multilevel process based on an intricate coordination of signal transduction pathways. In addition, it takes place in different subcellular compartments (cytosol, chloroplasts, mitochondria and peroxisomes), involving distinct second messengers (e.g., ROS and Ca^2+^) and protein-modifying enzymes (e.g., kinases and phosphatases) [4,5,6].

Stress-driven metabolic and transcriptional reprogramming of plant cells usually leads to a global response that ultimately affects plant physiology and development, mainly by means of phytohormones mediation, first of all abscisic acid (ABA) [5,7,8]. In angiosperms, the transition from the vegetative to the reproductive stage, referred to as flowering or heading, is crucial to ensure evolutionary success, and its timing and development are strongly regulated under unfavorable growing conditions. Indeed, exposure to abiotic stress during flower development would have deleterious effects on pollen viability and grain filling, threatening reproductive success and causing production losses in agricultural species [9,10]. Evolution and domestication have made plants adapt to abiotic stress by anticipating or delaying flowering, according to species-specific reproductive strategies [11]. In the model species *A. thaliana*, the cross-talk between the stress-signalling and the flowering regulatory pathways has been extensively reviewed, whereas, for other species, such as cultivated monocotyledons, information is more dispersed [12,13,14]. Cereals, belonging to the monocotyledon family of *Poaceae*, hold a great agricultural and economic impact, representing a primary food source for the world population and a major livestock feed [15,16].

Five cereal species sustain most of the human and animal nutritional needs and have therefore been extensively studied: wheat (*Triticum aestivum* L.), barley (*Hordeum vulgare* L.), rice (*Oryza sativa* L.), maize (*Zea mays* L.) and sorghum (*Sorghum bicolor* L.). According to the Food and Agriculture Organization, world production during 2020 reached 761, 157, 756, 1162 and 58 million tons for wheat, barley, rice, maize and sorghum, respectively [17]. Their productivity, as well as for other crops, relies on a successful transition from the vegetative to the reproductive and grain-filling phases [18].

Understanding cereal responses to different environmental events is crucial to boost their productivity and to ensure their resilience under stressful conditions. Floral initiation and its timing are tightly synchronized and controlled by genetic networks that integrate environmental cues as photoperiod and temperature [19]. In cultivated grasses, floral transition must occur at a very specific time (seasoning), in order to avoid deleterious stresses and foster a high grain yield [20,21].

Favorable flowering times differ between temperate and tropical cereals. The latter, including many rice varieties, preferentially flower under short day (SD) conditions to avoid pernicious high temperatures associated with the long-days (LD) season [22]. Conversely, temperate species generally flower under LD conditions (spring or summer) [21]. Amid the two categories, day-neutral crops, such as maize, do not have specific photoperiodic requirements for flowering, which is triggered by the activation of autonomous regulatory pathways [18].

Flowering-regulating genes can control many different traits, including seed formation and fertility. In cultivated monocotyledons, genetic variability of these genes has been associated with variations in productivity and plant survival under different climatic conditions [23,24,25,26,27]. This review aims at summarizing the currently available information to underline the molecular interconnection existing between photoperiodic flowering induction and the exposure to unfavorable atmospheric and soil conditions in cereals.

Drought, extreme temperatures and the excess of soil salinity are increasingly determining factors for productivity during the next decades, due to climate change [28]. Having a comprehensive overview of the genes and proteins involved in the control of heading time in cereals in response to specific abiotic stresses could help better define new biotechnological and breeding targets to improve their productivity in the field.

## 2. Molecular Regulators of Flowering Time in Monocotyledons

From the molecular point of view, monocotyledons share elements of an exclusive flowering-controlling pathway that integrates specific regulators with the *A. thaliana GIGANTEA-CONSTANS-FLOWERING LOCUS T (GI-CO-FT)* reference model [14,29].

In rice, *CO* homologue *Heading date 1* (*Hd1*) functionally differentiated in order to work in a counterposed manner (both as flowering repressor and promoter), depending on the photoperiod. Other important monocotyledon flowering regulators have different roles with respect to their *A. thaliana* homologues: for example, wheat and barley *Photoperiod 1* (*Ppd-1*), rice *PSEUDO-RESPONSE REGULATOR37* (*OsPRR37*) and sorghum *SbPRR37*, all flowering repressors, are homologous to *A. thaliana PRR37*, a pseudo-response regulator that has a role in the circadian clock, but not in flowering induction [30,31,32,33]. Similarly, temperate cereals *VERNALIZATION 1* (*VRN1*), an MADS-box gene involved in the vernalization process, is homologous to *A. thaliana APETALA 1 (AP1)*, whose role in flower development is only downstream of environmental induction [34,35]. On the other hand, grass-specific flowering regulators are numerous and include the homologous flowering repressors *Grain number, Plant Height, and Heading date1* (*Ghd7*) in rice and sorghum, barley vernalization regulator *VRN2*, and maize *CONSTANS, CONSTANS-LIKE AND TOC1 (ZmCCT)* [36,37,38].

In all cereals, the leaf-to-shoot mobilization of florigenic proteins, belonging to the phosphatidyl ethanolamine-binding protein (PEBP) family, and the formation of presumed florigen activation complexes, are the final output of photoperiodic flowering induction and the starting point for terminal shoot differentiation and floral organs development [39,40,41]. Cereals’ FT-like proteins include rice Hd3a and RFT1, *Z. mays* CENTRORADIALIS 8 (ZCN8) and 12, SbFT1, 8 and 10 in sorghum and HvFT/TaFT/VRN3 in barley and wheat [42,43,44,45,46].

## 3. Stress Factors and Flowering Response

### 3.1. Water Availability

In cereals, the effect of water availability on flowering time and yield broadly differs among species, being the result of the adaption to distinct edaphic and climatic conditions.

An excess of water in soil, known as water-logging, accelerates flowering in rice, but delays it in sorghum [47,48]. A general yield decrease, as a consequence of flooding, characterizes all rainfed crops, such as sorghum, maize and wheat [49,50]. Rice, instead, is often cultivated as a semiaquatic plant, requiring controlled land flooding, so that water excess is not normally considered as a stressful condition, unless it leads to submergence, thereby impairing photosynthesis and flowering [51].

Flooding is perceived by plant roots as a condition of general oxygen reduction (hypoxia), which alters the redox environment inside of the mitochondria [52]. In *A. thaliana* and cultivated monocotyledons, the systemic response to flooding mainly relies on the ethylene signalling pathway [52,53,54,55]. To the best of our knowledge, no in-depth studies on the molecular connection between the response to flooding and flowering induction in cereals have been conducted so far.

Conversely, much more data are available on the interplay between drought response and flowering, possibly because extreme drought events tend to represent a greater threat to agricultural production [56,57]. Prolonged water deficit causes a delay in the reproductive transition in most cereals [58,59]. In barley and wheat, drought-driven changes in flowering time and harvesting parameters are related to the genotypical seasonality [60]. These crops are cultivated in cold to temperate climates, where a winter season is clearly defined. Varieties that are sown in autumn, and harvested in summer, are called winter varieties, whereas those sown in spring, to be harvested in autumn, are called spring varieties and do not require vernalization [36]. A study conducted on barley has shown that spring and winter varieties react to drought in the pre-anthesis stages with a general heading delay, though the degree of such delay depends on the genotype and its photoperiodical requirements. However, harvest losses were reduced in early-flowering spring varieties [60].

The molecular link existing between floral induction and osmotic stress response has been deeply studied in *A. thaliana*, a facultative LD-flowering plant. A major role has been attributed to the clock component GI, positively regulating CO expression, which in turn promotes the transcription of the florigen FT. Under inductive photoperiodic conditions and drought stress, GI mediates the ABA-dependent mechanism of “drought escape”, directly or indirectly activating FT expression to accelerate flowering [61,62].

**Figure 1:**
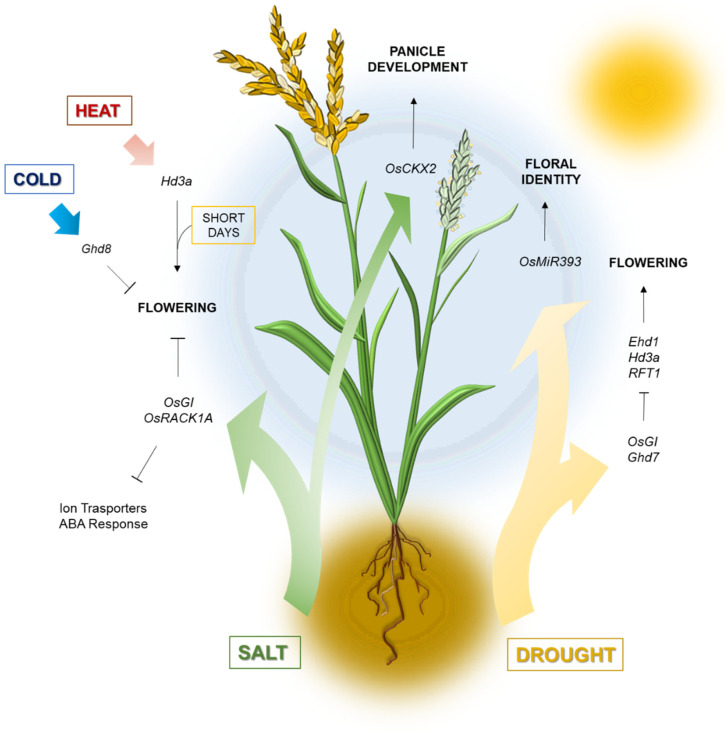
Rice flowering under abiotic stress. Drought, salt or extreme temperature stimuli converge on endogenous flowering regulators to modulate heading and floral organs development in *Oryza sativa*. Some of the major regulators involved in stress responses are indicated in italics; see text for additional details.

In rice, the transcription of the monocotyledon-specific floral promoter Early Heading Date 1 (Ehd1) and of the two florigens Hd3a and RFT1 are reduced by drought treatment under inductive photoperiodic conditions [63]. Rice GI homologue, OsGI, is a mild floral repressor controlling the expression of Hd1, an important flowering repressor under LD [64]. Rice *osgi* mutants are early flowering and show higher drought tolerance than the wildtype, as well as an upregulation of genes related to oxidative-stress response and protein stabilization [65,66,67,68]. Some researchers, however, have attributed to Ehd1 a more important role than OsGI in the integration of drought stimuli into the flowering pathway [63] (Figure 1).

Rice floral repressor Ghd7 has been reported to control various plant traits in addition to heading date, such as drought tolerance. Just as *osgi* mutants, *Ghd7* knockdown lines are early flowering and show increased drought tolerance, whereas *Ghd7*-overexpressing plants are more sensitive to water deprivation [69,70].

Overexpression of the *ZmCCT*, the maize homologue of rice *Ghd7*, delays flowering under LD and drought treatment, but also confers higher drought tolerance [71,72]. It could be hypothesized that Ghd7/ZmCCT participation in both flowering induction and drought stress response is conserved in monocotyledons, even if the specific gene function could vary between the two species.

In maize, ZmCCT regulates the expression of a number of genes belonging to the floral induction pathway and to the circadian clock, such as *ZmCOL9* and maize *CIRCADIAN CLOCK ASSOCIATED 1* (*ZmCCA1*), respectively. ZmCCA1 is an important clock component in maize and a positive regulator of a set of genes related to the general stress response, such as those encoding for the flagellin receptor *FLS2* or the MAP kinases *MKK1* and *2* [73,74,75,76]. *OsCCA1* has been recently associated with ABA signalling and the response to multiple abiotic stresses [77]. Conversely, the transcription of above-mentioned *Ghd7* is repressed both by drought and ABA treatments [69,70]. However, the interplay between these two genes under drought conditions needs yet to be clarified.

NUCLEAR FACTOR (NF-Y) transcription factors are another notable set of proteins that regulate heading date in cereals. NF-Y proteins are widespread among eucaryotes, controlling multiple developmental and stress-related processes through the formation of DNA-binding heterotrimers [78,79,80]. In flowering plants, the formation of NF-Y complexes is required to regulate the expression of florigenic genes. *A. thaliana* CO and rice Hd1 work as NF-YA subunits inside of NF-Y trimers, binding to *FT/Hd3a* promoter regions and activating gene transcription [81,82]. Interaction of NF-Y transcription factors with flowering and vernalization regulators (VRN2 and CO2) has also been reported in *Triticum monococcum* wheat [83].

*A. thaliana* NF-C3, NF-C4, and NF-C9 subunits have been shown to interact with ABA-responsive element-binding factors (ABFs) to enhance *SUPPRESSOR OF OVEREXPRESSOR OF CONSTANS1* (*SOC1*) transcription and so induce flowering under drought stress [84]. Maize transcription factor NF-YA3 interacts with FLOWERING PROMOTING FACTOR 1 (ZmFPF1) and the CCT protein ZmCO-like to promote flowering by binding to *ZmFT-like12* promoter [85]. Moreover, ZmNF-YA3 is able to bind to the promoters of a set of ABA-related transcription factors and to physically interact with MYC4, a protein belonging to the jasmonic acid (JA) signaling cascade [85].

In temperate cereals, barley Ppd-1 is an important hub for the integration of drought response into the control of flowering time. Indeed, *Ppd-1* is, together with the vernalization gene *VRN1*, the main quantitative trait associated with yield variability in relation to harsh climatic conditions in barley [27,86,87]. Under inductive photoperiodic conditions, Ppd-H1 promotes flowering by enhancing the expression of a set of downstream genes, such as *HvFT1*, *VRN1* and *MADS-box 3* (*BM3*). The barley *Ppd-1* promoter region contains ABA-responsive elements (ABREs), and gene transcription in enhanced by osmotic stress, similar to some *A. thaliana* PRRs [88,89]. Many high-latitudes barley varieties carry a *Ppd-1* version that encodes for a protein with a mutated CCT domain. Flowering is consequently delayed, generating a favorable trait for harvesting in colder climates. On the other hand, these varieties tend to be less drought tolerant: under suboptimal hydric conditions, the delay of heading date is larger and spike development is impaired [90]. These findings lead to thinking that Ppd-1 could mediate the response to water deficit during reproductive development. Flowering pathways in barley and wheat are very similar [31,32,91]; therefore, it is possible that a similar model is also valid for wheat, but this is still a speculation.

MiRNAs also have an important function in the regulation of drought response in plants [92,93]. In *A. thaliana*, GI promotes the processing of miR172, which targets a set of FT repressors to facilitate flowering during drought escape [29,94,95,96]. In rice, maize and barley, miR172s take part in the determination of floral identity and spikelet differentiation by targeting the transcripts of AP2 orthologs [97,98,99,100]. After drought treatment, miR172 target gene *Glossy15 (GL15)* is downregulated in maize [101]. However, there are no studies describing how miR172 might simultaneously connect drought stress and reproductive development networks in these crops.

OsmiR393 occupies an overlapping position, influencing both floral commitment and drought response, presumably through a modulation of auxin sensitivity in leaves and other organs. OsmiR393 negatively regulates auxin perception by targeting the auxin receptors OsTIR1 and OsAFB2, similarly to their *A. thaliana* homologues [102,103]. While a higher tillering rate and early flowering result from OsTIR1 and OsAFB2 repression, it is yet not known how this affects abiotic stress tolerance. Different research works have shown that higher miR393 levels are associated with lower salt and drought resistance both in rice and *A. thaliana*, but studies using a combined approach in rice are still missing [103,104,105] (Figure 1).

### 3.2. Temperature Extremes

Temperature thresholds for optimal plant growth largely vary among species. Wheat optimal range, out of which the plant perceives a temperature stress, is 17–23 °C [106]. The same parameter for rice and maize ranges between 13–35 °C and 6–42 °C, respectively [107]. Based on lethal temperatures, it is evident that wheat has a lower heat tolerance than rice, which is the most sensitive to low temperatures, whereas maize shows the largest optimal thermal range [91,106,107].

One of the most important features of temperate cereals is the adaptation to cold climates through the evolution of vernalization. Vernalization is a molecular mechanism ensuring that flowering takes place after the winter has passed, i.e., in warm and long days when the risk of frosting is lower. Vernalization is also present in dicotyledons, but it has most probably evolved independently more than once across evolution [108].

In *A. thaliana*, exposure to prolonged low temperatures and consequent upregulation of the vernalization gene *VERNALIZATION INSENSITIVE 3* (*VIN3*) results in the de-repression of flowering through epigenetic inactivation of the MADS-box transcription tactor *FLOWERING LOCUS C* (*FLC*), which otherwise inhibits the transition to the reproductive stage [109].

In vernalizing cereals, control of vernalization is based on a conserved set of three genes: *VRN1*, *VRN2* and *VRN3*. VRN2, a Ghd7 homologue, is a flowering repressor under LD, and suppresses *VRN3* expression before vernalization. After a long cold exposure, *VRN1* is upregulated, inactivating *VRN2* and de-repressing *VRN3*, which in turn enhances *VRN1* expression in a positive loop that brings the floral transition [14,46,110,111].

Although vernalization is required for proper life cycle both in *A. thaliana* and in temperate cereals, those species can also be sensitive to freezing temperatures. In *A. thaliana*, in fact, cold resistance of *gi* mutants is suppressed by knockout of *CYCLING DOF FACTORS* (*CDF*s). This suggests that GI and CDFs, which exert a combined control of *CO* expression, may also regulate the response to low temperatures, as demonstrated by transcriptomic data on differentially expressed genes in these mutants [112]. In *T. monococcum*, a set of *COLD-REGULATED* (COR) genes is regulated by VRN1, in a photoperiod-dependent way: they are upregulated under SD and downregulated under LD conditions [113].

Rice has adapted over history to cultivation at high latitudes, although it is a tropical plant in origin. Regardless of the photoperiod, low temperatures delay heading date in this species [114]. *Oryza japonica* subspecies carries a Ghd8 promoter allele that increases gene expression, presumably predisposing it to endure colder climates [26]. Ghd8 is a NF-YB subunit and a flowering repressor, and its overexpression has indeed been recently correlated to increased cold tolerance and transcription of cold responsive genes [26] (Figure 1).

It must be noted that increasing temperatures associated with climate change may represent a greater challenge to cereal production than frosting events in the upcoming decades [115].

In *A. thaliana*, high temperatures induce early flowering. Temperature-driven upregulation of FT in the leaf results from an interplay of ELF3, CO and the transcription factor PHYTOCHROME-INTERACTING FACTOR 4 (PIF4) [91,116,117,118].

In *Poaceae*, as for other types of stress, the scenario is not uniform [60,119,120,121]. In *Brachypodium dystachion*, a monocotyledon model species, heading is delayed at temperatures that are both lower or higher than the optimal one, although the intensity of the phenotype is accession-dependent [122].

In rice, higher temperatures accelerate heading under SD by enhancing *Hd3a* expression, but this does not happen under LD conditions, indicating that temperature-mediated flowering promotion is dependent on the photoperiod [114,123].

In barley, a shift to higher temperatures during the vegetive phase promotes flowering under inductive conditions (LD) and delays it under non-inductive ones (SD). This response pattern is dependent on the MADS-box floral repressor HvODDSOC2 (HvOS2), which is upregulated at high temperatures under SD condition. *OS2* genes are specific of grasses and show only a weak similarity to *A. thaliana SOC1*. Both wheat, barley and *B. distachyon OS2* orthologues are downregulated by cold treatment, suggesting that convergence of both photoperiod- and temperature-dependent flowering regulating functions might be phylogenetically conserved [124,125,126].

Barley differential response to rising temperatures in terms of heading date has been also attributed to already-mentioned *Ppd-1*, as well as to *HvELF3*: mutation of the former delays flowering under high temperatures, while mutation in the latter, a repressor of Ppd-1, accelerates it [127]. Because Ppd-1 is upstream of VRN1 and HvODDSOC2, it could be inferred that an entire section of the flowering induction pathway is modulated in response to temperature variations to orient heading date in barley.

Finally, coordination between photoperiod and temperature sensing to control floral induction is mediated by the lectin-like gene *TaVER2* and its barley homologue. The transcripts of the two genes increase with vernalization but decrease under high temperature and SD conditions [128].

A major risk of heat stress during reproductive development is permanent damage to floral organs. Maximum anthesis temperatures reach 32 °C in wheat and 37 °C in rice and maize: beyond these points, yield losses and seed sterility are substantial [91,106,107]. Many monocotyledons, such as wheat, sorghum and millet, have adapted flower opening in order to occur in early morning or late evening, when temperatures are lower [129]. The qEMF locus has been identified as responsible for advancing the flower opening to early morning in *Oryza officinalis* and represents a trait of agronomical interest to reduce heat-born damages to floral organs in domesticated rice [130].

### 3.3. Soil Salinity

Soil salinity is an especially relevant problem in coastal agricultural areas, and its magnitude is expected to increase due to the rising of sea levels and saltwater intrusion. Among other factors, waste-water contamination and excessive fertilization can also cause soil salinization [131].

Studies on halophytic plants have shown that tolerance to NaCl often correlates with a higher ability to cope with heavy metal soil pollution, a phenomenon that is of growing environmental interest. In fact, salinity stress has both an osmotic and an ionic component, and at least part of plant tolerance processes is expected to be common between these two types of abiotic stress [132,133,134].

Among cereals, rice exhibits a high sensitivity to salt, which delays heading and impairs the development of reproductive organs [135]. For its biological and agronomical interest, salt tolerance mechanisms in rice have been the subject of extensive research [55,135,136,137,138].

*A. thaliana* GI and its rice homologue OsGI have both been correlated to NaCl homeostasis. In *A. thaliana*, GI degradation under salt stress triggers a post-translational regulation pathway that activates the ion transporter SOS1, increasing cell tolerance to salt excess [139]. Analogously, OsGI is presumably targeted by a putative rice evening complex, composed by the rice clock proteins EARLY FLOWERING4 A (OsELF4A), OsELF3 and LUX ARRHYTHMO (OsLUX). Single mutants of the three complex components exhibit reduced salt tolerance and delayed heading date, while *osgi* mutants show the opposite phenotype. Higher salt tolerance in the latter coincides with the upregulation of genes encoding for ion transporters, such as *OsHAK1* and *OsHAK5* [140].

In rice, OsPRR73, another clock component, has lastly been described as a positive regulator of salt tolerance in rice, yet with no link to flowering [141]. On the other hand, rice *RECEPTOR FOR ACTIVATED C KINASE 1 A* (OsRACK1A), a clock-regulated gene, delays heading while it suppresses salt response by repression of stress-related genes [142]. *OsRACK1A* has an expression peak during the day, but night transcript levels increase during salt stress. Protein stability could be controlled post-translationally, since it is phosphorylated under ABA treatment and drought conditions. Curiously, OsRACK1A accumulation peak coincides with that of OsGI, at 8–10 h from day start, suggesting that the two proteins could undergo a common diurnal control [142] (Figure 1, Table 1).

As for other stresses, transcriptional control of protein-coding genes in response to salinity does not represent the whole scenario. The role of miRNAs in salt stress tolerance has been elucidated in rice and maize. Previously mentioned OsmiR393 decreases tolerance to salt and alkaline stress, similarly to OsmiR396, which, on the other hand, has not been related to any flowering trait [143]. In maize, miR164s downregulation after salt treatment leads to enhanced expression of their degradation targets, which include members of the NAC transcription factor family. MiR164s are involved in the regulation of many developmental processes in plants, mainly by defining organ boundaries during meristem differentiation [117,118,144]. Distinct studies confirm the importance of miR164s and their target genes to define meristem boundaries also in rice, as well as their function in response to heavy metals and osmotic stress [120,121,140].

It can be hypothesized that miR164s work as a bridge between organ specification and abiotic stress response. The existence of a link between these two seemingly separate areas of plant biology is corroborated by a growing number of publications [145,146]. In rice, knockdown of OsCKX2, an inflorescence meristem-specific cytokinin oxidase, increases cytokinin levels during panicle development and confers high salt stress tolerance. Moreover, since OsCKX2 is a negative regulator of branching, productivity-related parameters, such as branch and grain number, are enhanced in RNAi lines, to a greater extent under salinity stress [147] (Figure 1). Adaptation to high salt concentrations during flower development, far downstream of the photoperiodic induction pathway, is essential to overcome toxic effects on the development of reproductive organs, which are well documented in crops [138,148,149].

Unfortunately, in temperate cereals, the molecular players of salt stress response in relation to flowering regulation have not been studied in depth. In wheat and barley, NaCl is excluded from the floral apex under high salinity conditions. This notwithstanding, reproductive development and productivity are equally affected: salt-treated plants are early flowering and produce a reduced number of spikelets and grains [148] (Figure 1). Field studies on different barley accessions point at an existing correlation between photoperiodic responsiveness and tolerance to abiotic stresses, such as drought and salinity, in association with the allelic variability of the flowering time genes *Ppd-1, Sdw1*, *VRN1* and *VRN3* [27]. It is highly probable that these genes’ functions are transversal to different physiological processes, as demonstrated in the case of heading and drought stress response.

## 4. Conclusions and Perspectives

In this review, the role of important monocotyledon regulators of abiotic stress response and flowering induction has been addressed (Table 1).

Although the focus here has been mainly on photoperiodic-responsive genes controlling the timing of the reproductive phase, a global response to abiotic stress vehiculated by downstream events, such the formation of the florigen activation complexes in the shoot apical meristem prior to the reproductive differentiation, may take place.

Florigens accomplish their regulatory function through the interaction with a group of bZip transcription factors, homologous to *A. thaliana* FD [41,42,46,150].

Among the bZIP family, many TFs are linked to ABA-mediated abiotic stress response [151]. In rice, for example, OsbZIP72 is a positive regulator of ABA response and drought tolerance [152]. In addition, OsbZIP46 overexpression improves drought tolerance, participating in ABA response [153]. OsbZIP23, 66 and 72 are, instead, involved in the regulation of ABA-mediated seed germination via interaction with OsMFT2 (MOTHER OF FT AND TERMINAL FLOWER 1), which belongs to the PEBP family [154].

In *A. thaliana,* bZIPs associated with ABA signalling have been also shown to participate in flowering regulation upstream of the vernalization gene FLC [155]. Future investigations in this direction could help with dissecting the role of bZIP transcription factors in vernalization and abiotic stress response in temperate cereals.

Finally, a relevant position between flowering control and abiotic stress response might be taken by post-translational modifications (PTMs). PTMs are an essential component of the complex interaction network on which plants rely to adapt to environmental stresses. Through the addition of small chemical groups, PTMs regulate protein subcellular localization and/or interactions with other proteins [4,156,157,158]. Ubiquitination, which is crucial to control proteins abundance in cells, has been reported to contribute to the acquisition of stress tolerance in plants [159]. In rice, for example, the role of E3-ubiquitin ligases, and of their interacting proteins, in association with drought response has been largely studied. Nevertheless, no sufficient data are available to establish a link with flowering [160,161].

Reversible protein phosphorylation induced by abiotic stresses such as salinity or drought is known to influence cereal productivity by causing growth delays or fertility impairment. Moreover, phosphoproteomics analyses have identified several kinases or kinase-interacting proteins that are involved in male-sterility processes [157,162,163,164,165].

**Table 1:** Main flowering regulators of *Poaceae* integrating abiotic stress signalling into the heading control and flower developmental pathways.

Regulator	Species	Stress	Role in Flowering	Reference
OsGI	Rice	Drought, salinity	Repressor	[64,68,140]
Ehd1	Rice	Drought	Promoter	[63]
OsELF4a	Rice	Salinity	Promoter	[140]
OsELF3	Rice	Salinity	Promoter	[140]
OsLUX	Rice	Salinity	Promoter	[140]
Ghd7	Rice	Drought	Repressor	[70]
OsRACK1A	Rice	Salinity	Repressor	[142]
OsCKX2	Rice	Salinity	Panicle Development	[147]
Ghd8	Rice	Cold	Repressor	[26]
miR393	Rice	Drought, salinity	Promoter	[102,103]
miR172	Rice, maize, barley	Drought	Panicle Development	[97,99,101,166]
ZmCCT	Maize	Drought	Repressor	[71,72]
NF-YA3	Maize	Drought	Promoter	[85]
miR164	Maize, rice	Salinity, drought	Meristem differentiation	[167]
Ppd-1	Barley	Drought, heat	Promoter	[90,127]
HvVRN1	Barley	Heat	Vernalization/Promoter	[125,126,127]
HvODDSOC2	Barley	Heat	Repressor	[125,126]
VRN1	*T. monococcum*	Cold	Vernalization/Promoter	[113]
BdVIL4	*B. dystachion*	Heat	Vernalization/Promoter	[168]

The issue of soil nutrient availability in relation to plant reproductive development has not been addressed in this review. Evidence exists that the plant nutritional status influences flowering and grain-filling phases in cereals [91]. Even if soil nutrient imbalance is certainly a major source of abiotic stress for plants, specific deficiencies can be often handled with cultivation techniques. Obviously, these applications are not that effective in mitigating unpredictable weather episodes and extreme environmental phenomena.

It is a fact that climate change has negative effects on cereals lifecycle and productivity, and that the frequency of severe heat, drought, or salinity events is growing at a high pace. Considerable yield decreases and geographically determined changes in crop phenology are amongst the expected effects of such extreme events [28].

Considering the current scenario of constantly changing climate conditions, a more detailed understanding of how abiotic stress variables affect the molecular control of flowering in cereals is important for their future biotechnological optimization and to improve productivity in the field.

## Data Availability

No new data were created or analyzed in this study. Data sharing is not applicable to this article.

## References

[1] Kim J.M., To T.K., Nishioka T., Seki M. (2010). Chromatin Regulation Functions in Plant Abiotic Stress Responses. Plant Cell Environ..

[2] Urano K., Kurihara Y., Seki M., Shinozaki K. (2010). ‘Omics’ Analyses of Regulatory Networks in Plant Abiotic Stress Responses. Curr. Opin. Plant Biol..

[3] Chang Y.-N., Zhu C., Jiang J., Zhang H., Zhu J.-K., Duan C.-G. (2020). Epigenetic Regulation in Plant Abiotic Stress Responses. J. Integr. Plant Biol..

[4] Zhang H., Zhu J., Gong Z., Zhu J.-K. (2022). Abiotic Stress Responses in Plants. Nat. Rev. Genet..

[5] Danquah A., de Zelicourt A., Colcombet J., Hirt H. (2014). The Role of ABA and MAPK Signaling Pathways in Plant Abiotic Stress Responses. Biotechnol. Adv..

[6] Mittler R., Zandalinas S.I., Fichman Y., van Breusegem F. (2022). Reactive Oxygen Species Signalling in Plant Stress Responses. Nat. Rev. Mol. Cell Biol..

[7] Cramer G.R., Urano K., Delrot S., Pezzotti M., Shinozaki K. (2011). Effects of Abiotic Stress on Plants: A Systems Biology Perspective. BMC Plant Biol..

[8] Fahad S., Nie L., Chen Y., Wu C., Xiong D., Saud S., Hongyan L., Cui K., Huang J., Lichtfouse E. (2015). Crop Plant Hormones and Environmental Stress. Sustainable Agriculture Reviews: Volume 15.

[9] Zinn K.E., Tunc-Ozdemir M., Harper J.F. (2010). Temperature Stress and Plant Sexual Reproduction: Uncovering the Weakest Links. J. Exp. Bot..

[10] De Storme N., Geelen D. (2014). The Impact of Environmental Stress on Male Reproductive Development in Plants: Biological Processes and Molecular Mechanisms. Plant Cell Environ..

[11] Cho L.-H., Yoon J., An G. (2017). The Control of Flowering Time by Environmental Factors. Plant J..

[12] Barnabás B., Jäger K., Fehér A. (2008). The Effect of Drought and Heat Stress on Reproductive Processes in Cereals. Plant Cell Environ..

[13] Park H.J., Kim W.-Y., Pardo J.M., Yun D.-J., Jeon K.W., Galluzzi L. (2016). Chapter Eight–Molecular Interactions Between Flowering Time and Abiotic Stress Pathways. International Review of Cell and Molecular Biology.

[14] Brambilla V., Gómez-Ariza J., Cerise M., Fornara F. (2017). The Importance of Being on Time: Regulatory Networks Controlling Photoperiodic Flowering in Cereals. Front. Plant Sci..

[15] Awika J.M. (2011). Major Cereal Grains Production and Use around the World. Advances in Cereal Science: Implications to Food Processing and Health Promotion.

[16] Sarwar H. (2013). The Importance of Cereals (Poaceae: Gramineae) Nutrition in Human Health: A Review. J. Cereals Oilseeds.

[17] FAOSTAT (2022). https://www.fao.org/faostat/en/#home.

[18] Hill C.B., Li C. (2016). Genetic Architecture of Flowering Phenology in Cereals and Opportunities for Crop Improvement. Front. Plant Sci..

[19] Andrés F., Coupland G. (2012). The Genetic Basis of Flowering Responses to Seasonal Cues. Nat. Rev. Genet..

[20] McCorriston J., Hole F. (1991). The Ecology of Seasonal Stress and the Origins of Agriculture in the Near East. Am. Anthropol..

[21] Greenup A., Peacock W.J., Dennis E.S., Trevaskis B. (2009). The Molecular Biology of Seasonal Flowering-Responses in Arabidopsis and the Cereals. Ann. Bot..

[22] Yano M., Kojima S., Takahashi Y., Lin H., Sasaki T. (2001). Genetic Control of Flowering Time in Rice, a Short-Day Plant. Plant Physiol..

[23] Zhang J., Zhou X., Yan W., Zhang Z., Lu L., Han Z., Zhao H., Liu H., Song P., Hu Y. (2015). Combinations of the Ghd7, Ghd8 and Hd1 Genes Largely Define the Ecogeographical Adaptation and Yield Potential of Cultivated Rice. New Phytol..

[24] Zhang B., Liu H., Qi F., Zhang Z., Li Q., Han Z., Xing Y. (2019). Genetic Interactions Among Ghd7, Ghd8, OsPRR37 and Hd1 Contribute to Large Variation in Heading Date in Rice. Rice.

[25] Klein R.R., Miller F.R., Dugas D.V., Brown P.J., Burrell A.M., Klein P.E. (2015). Allelic Variants in the PRR37 Gene and the Human-Mediated Dispersal and Diversification of Sorghum. Theor. Appl. Genet..

[26] Wang P., Xiong Y., Gong R., Yang Y., Fan K., Yu S. (2019). A Key Variant in the Cis-Regulatory Element of Flowering Gene Ghd8 Associated with Cold Tolerance in Rice. Sci. Rep..

[27] Wiegmann M., Maurer A., Pham A., March T.J., Al-Abdallat A., Thomas W.T.B., Bull H.J., Shahid M., Eglinton J., Baum M. (2019). Barley Yield Formation under Abiotic Stress Depends on the Interplay between Flowering Time Genes and Environmental Cues. Sci. Rep..

[28] Fatima Z., Ahmed M., Hussain M., Abbas G., Ul-Allah S., Ahmad S., Ahmed N., Ali M.A., Sarwar G., Haque E.U. (2020). The Fingerprints of Climate Warming on Cereal Crops Phenology and Adaptation Options. Sci. Rep..

[29] Mizoguchi T., Wright L., Fujiwara S., Cremer F., Lee K., Onouchi H., Mouradov A., Fowler S., Kamada H., Putterill J. (2005). Distinct Roles of GIGANTEA in Promoting Flowering and Regulating Circadian Rhythms in Arabidopsis. Plant Cell.

[30] Murakami M., Matsushika A., Ashikari M., Yamashino T., Mizuno T. (2005). Circadian-Associated Rice Pseudo Response Regulators (OsPRRs): Insight into the Control of Flowering Time. Biosci. Biotechnol. Biochem..

[31] Turner A., Beales J., Faure S., Dunford R., Laurie D. (2005). Botany: The Pseudo-Response Regulator Ppd-H1 Provides Adaptation to Photoperiod in Barley. Science.

[32] Kitagawa S., Shimada S., Murai K. (2012). Effect of Ppd-1 on the Expression of Flowering-Time Genes in Vegetative and Reproductive Growth Stages of Wheat. Genes Genet. Syst..

[33] Yang L., Liu T., Li B., Sui Y., Chen J., Shi J., Wing R.A., Chen M. (2012). Comparative Sequence Analysis of the Ghd7 Orthologous Regions Revealed Movement of Ghd7 in the Grass Genomes. PLoS ONE.

[34] Yan L., Loukoianov A., Tranquilli G., Helguera M., Fahima T., Dubcovsky J. (2003). Positional Cloning of the Wheat Vernalization Gene VRN1. Proc. Natl. Acad. Sci. USA.

[35] Gregis V., Sessa A., Dorca-Fornell C., Kater M.M. (2009). The Arabidopsis Floral Meristem Identity Genes AP1, AGL24 and SVP Directly Repress Class B and C Floral Homeotic Genes. Plant J..

[36] Distelfeld A., Li C., Dubcovsky J. (2009). Regulation of Flowering in Temperate Cereals. Curr. Opin. Plant Biol..

[37] Zhang L., Li Q., Dong H., He Q., Liang L., Tan C., Han Z., Yao W., Li G., Zhao H. (2015). Three CCT Domain-Containing Genes Were Identified to Regulate Heading Date by Candidate Gene-Based Association Mapping and Transformation in Rice. Sci. Rep..

[38] Murphy R.L., Morishige D.T., Brady J.A., Rooney W.L., Yang S., Klein P.E., Mullet J.E. (2014). Ghd7 (Ma6) Represses Sorghum Flowering in Long Days: Ghd7 Alleles Enhance Biomass Accumulation and Grain Production. Plant Genome.

[39] Karlgren A., Gyllenstrand N., Källman T., Sundström J.F., Moore D., Lascoux M., Lagercrantz U. (2011). Evolution of the PEBP Gene Family in Plants: Functional Diversification in Seed Plant Evolution. Plant Physiol..

[40] Taoka K., Ohki I., Tsuji H., Kojima C., Shimamoto K. (2013). Structure and Function of Florigen and the Receptor Complex. Trends Plant Sci..

[41] Li C., Lin H., Dubcovsky J. (2015). Factorial Combinations of Protein Interactions Generate a Multiplicity of Florigen Activation Complexes in Wheat and Barley. Plant J..

[42] Lazakis C.M., Coneva V., Colasanti J. (2011). ZCN8 Encodes a Potential Orthologue of Arabidopsis FT Florigen That Integrates Both Endogenous and Photoperiod Flowering Signals in Maize. J. Exp. Bot..

[43] Wolabu T.W., Zhang F., Niu L., Kalve S., Bhatnagar-Mathur P., Muszynski M.G., Tadege M. (2016). Three FLOWERING LOCUS T-like Genes Function as Potential Florigens and Mediate Photoperiod Response in Sorghum. New Phytol..

[44] Castelletti S., Coupel-Ledru A., Granato I., Palaffre C., Cabrera-Bosquet L., Tonelli C., Nicolas S.D., Tardieu F., Welcker C., Conti L. (2020). Maize Adaptation across Temperate Climates Was Obtained via Expression of Two Florigen Genes. PLoS Genet..

[45] Yan L., Fu D., Li C., Blechl A., Tranquilli G., Bonafede M., Sanchez A., Valarik M., Yasuda S., Dubcovsky J. (2006). The wheat and barley vernalization gene *VRN3* is an orthologue of *FT*. Proc. Natl. Acad. Sci. USA.

[46] Li C., Dubcovsky J. (2008). Wheat FT Protein Regulates VRN1 Transcription through Interactions with FDL2. Plant J..

[47] Juraimi A., Ahmad Hamdani M.S., Begum M., Anuar A.R., Azmi M. (2009). Influence of Flooding Intensity and Duration on Rice Growth and Yield. Pertanika J. Trop. Agric. Sci..

[48] Haussmann B.I.G., Fred Rattunde H., Weltzien-Rattunde E., Traoré P.S.C., vom Brocke K., Parzies H.K. (2012). Breeding Strategies for Adaptation of Pearl Millet and Sorghum to Climate Variability and Change in West Africa. J. Agron. Crop. Sci..

[49] LI S., Tompkins A.M., Lin E., Ju H. (2016). Simulating the Impact of Flooding on Wheat Yield—Case Study in East China. Agric. For. Meteorol..

[50] Promkhambut A., Polthanee A., Akkasaeng C., Younger A. (2011). Growth, Yield and Aerenchyma Formation of Sweet and Multipurpose Sorghum (*Sorghum bicolor* L. Moench) as Affected by Flooding at Different Growth Stages. Aust. J. Crop Sci..

[51] Lin C., Zhu T., Peralta Ogorek L., Wang Y., Sauter M., Pedersen O. (2022). The Pyramiding of Three Key Root Traits Aid Breeding of Flood-Tolerant Rice. Plants.

[52] Dat J.F., Capelli N., Folzer H., Bourgeade P., Badot P.-M. (2004). Sensing and Signalling during Plant Flooding. Plant Physiol. Biochem..

[53] Bragina T.V., Rodionova N.A., Grinieva G.M. (2003). Ethylene Production and Activation of Hydrolytic Enzymes during Acclimation of Maize Seedlings to Partial Flooding. Russ. J. Plant Physiol..

[54] Larsen O., Nilsen H.-G., Aarnes H. (1986). Response of Young Barley Plants to Waterlogging, as Related to Concentration of Ethylene and Ethane. J. Plant Physiol..

[55] Steffens B. (2014). The Role of Ethylene and ROS in Salinity, Heavy Metal, and Flooding Responses in Rice. Front. Plant Sci..

[56] Shitsukawa N., Ikari C., Shimada S., Kitagawa S., Sakamoto K., Saito H., Ryuto H., Fukunishi N., Abe T., Takumi S. (2007). The Einkorn Wheat (*Triticum Monococcum*) Mutant, *Maintained Vegetative Phase*, Is Caused by a Deletion in the *VRN1* Gene. Genes Genet. Syst..

[57] Shavrukov Y., Kurishbayev A., Jatayev S., Shvidchenko V., Zotova L., Koekemoer F., Groot S., Soole K., Langridge P. (2017). Early Flowering as a Drought Escape Mechanism in Plants: How Can It Aid Wheat Production?. Front. Plant Sci..

[58] Abrecht D.G., Carberry P.S. (1993). The Influence of Water Deficit Prior to Tassel Initiation on Maize Growth, Development and Yield. Field Crops Res..

[59] McMaster G.S., Wilhelm W.W. (2003). Phenological Responses of Wheat and Barley to Water and Temperature: Improving Simulation Models. J. Agric. Sci..

[60] Al-Ajlouni Z.I., Al-Abdallat A.M., Al-Ghzawi A.L.A., Ayad J.Y., Abu Elenein J.M., Al-Quraan N.A., Baenziger P.S. (2016). Impact of Pre-Anthesis Water Deficit on Yield and Yield Components in Barley (Hordeum Vulgare L.) Plants Grown under Controlled Conditions. Agronomy.

[61] Jose J., Bánfalvi Z. (2019). The Role of GIGANTEA in Flowering and Abiotic Stress Adaptation in Plants. Columella J. Agric. Environ. Sci..

[62] Mishra P., Panigrahi K.C. (2015). GIGANTEA—An Emerging Story. Front. Plant Sci..

[63] Galbiati F., Chiozzotto R., Locatelli F., Spada A., Genga A., Fornara F. (2016). Hd3a, RFT1 and Ehd1 Integrate Photoperiodic and Drought Stress Signals to Delay the Floral Transition in Rice. Plant Cell Environ..

[64] Hayama R., Yokoi S., Tamaki S., Yano M., Shimamoto K. (2003). Adaptation of Photoperiodic Control Pathways Produces Short-Day Flowering in Rice. Nature.

[65] Xiong J., Zhang L., Fu G., Yang Y., Zhu C., Tao L. (2012). Drought-Induced Proline Accumulation Is Uninvolved with Increased Nitric Oxide, Which Alleviates Drought Stress by Decreasing Transpiration in Rice. J. Plant Res..

[66] Wang W., Vinocur B., Shoseyov O., Altman A. (2004). Role of Plant Heat-Shock Proteins and Molecular Chaperones in the Abiotic Stress Response. Trends Plant Sci..

[67] Cao S., Jiang S., Zhang R. (2006). The Role of GIGANTEA Gene in Mediating the Oxidative Stress Response and in Arabidopsis. Plant Growth Regul..

[68] Li S., Yue W., Wang M., Qiu W., Zhou L., Shou H. (2016). Mutation of OsGIGANTEA Leads to Enhanced Tolerance to Polyethylene Glycol-Generated Osmotic Stress in Rice. Front. Plant Sci..

[69] Xue W., Xing Y., Weng X., Zhao Y., Tang W., Wang L., Zhou H., Yu S., Xu C., Li X. (2008). Natural Variation in Ghd7 Is an Important Regulator of Heading Date and Yield Potential in Rice. Nat. Genet..

[70] Weng X., Wang L., Wang J., Hu Y., Du H., Xu C., Xing Y., Li X., Xiao J., Zhang Q. (2014). Grain Number, Plant Height, and Heading Date7 Is a Central Regulator of Growth, Development, and Stress Response. Plant Physiol..

[71] Su H., Liang J., Abou-Elwafa S.F., Cheng H., Dou D., Ren Z., Xie J., Chen Z., Gao F., Ku L. (2021). ZmCCT Regulates Photoperiod-Dependent Flowering and Response to Stresses in Maize. BMC Plant Biol..

[72] Ku L., Tian L., Su H., Wang C., Wang X., Wu L., Shi Y., Li G., Wang Z., Wang H. (2016). Dual Functions of the ZmCCT-Associated Quantitative Trait Locus in Flowering and Stress Responses under Long-Day Conditions. BMC Plant Biol..

[73] Shi Y., Zhao X., Guo S., Dong S., Wen Y., Han Z., Jin W., Chen Y. (2020). ZmCCA1a on Chromosome 10 of Maize Delays Flowering of Arabidopsis Thaliana. Front. Plant Sci..

[74] Song K., Kim H., Shin S., Kim K.-H., Moon J.-C., Kim J.Y., Lee B.-M. (2017). Transcriptome Analysis of Flowering Time Genes under Drought Stress in Maize Leaves. Front. Plant Sci..

[75] Yu Y., Shi J., Li X., Liu J., Geng Q., Shi H., Ke Y., Sun Q. (2018). Transcriptome Analysis Reveals the Molecular Mechanisms of the Defense Response to Gray Leaf Spot Disease in Maize. BMC Genom..

[76] Shi F., Zhang Y., Wang K., Meng Q., Liu X., Ma L., Li Y., Liu J., Ma L. (2018). Expression Profile Analysis of Maize in Response to Setosphaeria Turcica. Gene.

[77] Wei H., Xu H., Su C., Wang X., Wang L. (2022). Rice CIRCADIAN CLOCK ASSOCIATED 1 Transcriptionally Regulates ABA Signaling to Confer Multiple Abiotic Stress Tolerance. Plant Physiol..

[78] Li G., Zhao H., Wang L., Wang Y., Guo X., Baohua X. (2018). The Animal Nuclear Factor Y: An Enigmatic and Important Heterotrimeric Transcription Factor. Am. J. Cancer Res..

[79] Swain S., Myers Z.A., Siriwardana C.L., Holt B.F. (2017). The Multifaceted Roles of NUCLEAR FACTOR-Y in Arabidopsis Thaliana Development and Stress Responses. Biochim. Biophys. Acta Gene Regul. Mech..

[80] Laloum T., De Mita S., Gamas P., Baudin M., Niebel A. (2013). CCAAT-Box Binding Transcription Factors in Plants: Y so Many?. Trends Plant Sci..

[81] Gnesutta N., Kumimoto R.W., Swain S., Chiara M., Siriwardana C., Horner D.S., Holt B.F., Mantovani R. (2017). CONSTANS Imparts DNA Sequence Specificity to the Histone Fold NF-YB/NF-YC Dimer. Plant Cell.

[82] Zhu S., Wang J., Cai M., Zhang H., Wu F., Xu Y., Li C., Cheng Z., Zhang X., Guo X. (2017). The OsHAPL1-DTH8-Hd1 Complex Functions as the Transcription Regulator to Repress Heading Date in Rice. J. Exp. Bot..

[83] Li C., Distelfeld A., Comis A., Dubcovsky J. (2011). Wheat Flowering Repressor VRN2 and Promoter CO2 Compete for Interactions with NUCLEAR FACTOR-Y Complexes. Plant J..

[84] Hwang K., Susila H., Nasim Z., Jung J.-Y., Ahn J.H. (2019). Arabidopsis ABF3 and ABF4 Transcription Factors Act with the NF-YC Complex to Regulate SOC1 Expression and Mediate Drought-Accelerated Flowering. Mol. Plant.

[85] Su H., Cao Y., Ku L., Yao W., Cao Y., Ren Z., Dou D., Wang H., Ren Z., Liu H. (2018). Dual Functions of ZmNF-YA3 in Photoperiod-Dependent Flowering and Abiotic Stress Responses in Maize. J. Exp. Bot..

[86] Von Korff M., Grando S., Del Greco A., This D., Baum M., Ceccarelli S. (2008). Quantitative Trait Loci Associated with Adaptation to Mediterranean Dryland Conditions in Barley. Theor. Appl. Genet..

[87] Rollins J.A., Drosse B., Mulki M.A., Grando S., Baum M., Singh M., Ceccarelli S., von Korff M. (2013). Variation at the Vernalisation Genes Vrn-H1 and Vrn-H2 Determines Growth and Yield Stability in Barley (Hordeum Vulgare) Grown under Dryland Conditions in Syria. Theor. Appl. Genet..

[88] Habte E., Müller L.M., Shtaya M., Davis S.J., Von Korff M. (2013). Osmotic Stress at the Barley Root Affects Expression of Circadian Clock Genes in the Shoot. Plant Cell Environ..

[89] Nakamichi N., Takao S., Kudo T., Kiba T., Wang Y., Kinoshita T., Sakakibara H. (2016). Improvement of Arabidopsis Biomass and Cold-, Drought-, and Salinity-Stress Tolerance by Modified Circadian Clock-Associated PSEUDO-RESPONSE REGULATORs. Plant Cell Physiol..

[90] Gol L., Haraldsson E.B., von Korff M. (2021). Ppd-H1 Integrates Drought Stress Signals to Control Spike Development and Flowering Time in Barley. J. Exp. Bot..

[91] Gol L., Tomé F., von Korff M. (2017). Floral Transitions in Wheat and Barley: Interactions between Photoperiod, Abiotic Stresses, and Nutrient Status. J. Exp. Bot..

[92] Guleria P., Goswami D., Mahajan M., Kumar V., Bhardwaj J., Yadav S. (2012). MicroRNAs and Their Role in Plants during Abiotic Stresses. Environmental Adaptations and Stress Tolerance of Plants in the Era of Climate Change.

[93] Chen Z., Li F., Yang S., Dong Y., Yuan Q., Wang F., Li W., Jiang Y., Jia S., Pei X. (2014). Identification and Functional Analysis of Flowering Related MicroRNAs in Common Wild Rice (*Oryza Rufipogon* Griff.). PLoS ONE.

[94] Mathieu J., Yant L.J., Mürdter F., Küttner F., Schmid M. Repression of flowering by the miR172 target SMZ. PLoS Biol..

[95] Jung J.-H., Seo Y.-H., Seo P.J., Reyes J.L., Yun J., Chua N.-H., Park C.-M. (2007). The GIGANTEA-Regulated MicroRNA172 Mediates Photoperiodic Flowering Independent of CONSTANS in Arabidopsis. Plant Cell.

[96] Han Y., Zhang X., Wang W., Wang Y., Ming F. (2013). The Suppression of WRKY44 by GIGANTEA-MiR172 Pathway Is Involved in Drought Response of Arabidopsis Thaliana. PLoS ONE.

[97] Zhu Q.-H., Upadhyaya N.M., Gubler F., Helliwell C.A. (2009). Over-Expression of MiR172 Causes Loss of Spikelet Determinacy and Floral Organ Abnormalities in Rice (Oryza Sativa). BMC Plant Biol..

[98] Chuck G., Meeley R., Irish E., Sakai H., Hake S. (2007). The Maize Tasselseed4 MicroRNA Controls Sex Determination and Meristem Cell Fate by Targeting Tasselseed6/Indeterminate Spikelet1. Nat. Genet..

[99] Brown R., Bregitzer P. (2011). A Ds Insertional Mutant of a Barley MiR172 Gene Results in Indeterminate Spikelet Development. Crop. Sci..

[100] Nair S.K., Wang N., Turuspekov Y., Pourkheirandish M., Sinsuwongwat S., Chen G., Sameri M., Tagiri A., Honda I., Watanabe Y. (2010). Cleistogamous Flowering in Barley Arises from the Suppression of MicroRNA-Guided HvAP2 MRNA Cleavage. Proc. Natl. Acad. Sci. USA.

[101] Kong Y.M., Elling A.A., Chen B., Deng X.W. (2010). Differential Expression of MicroRNAs in Maize Inbred and Hybrid Lines during Salt and Drought Stress. Am. J. Plant Sci..

[102] Si-Ammour A., Windels D., Arn-Bouldoires E., Kutter C., Ailhas J., Meins F.J., Vazquez F. (2011). MiR393 and Secondary SiRNAs Regulate Expression of the TIR1/AFB2 Auxin Receptor Clade and Auxin-Related Development of Arabidopsis Leaves. Plant Physiol..

[103] Xia K., Wang R., Ou X., Fang Z., Tian C., Duan J., Wang Y., Zhang M. (2012). OsTIR1 and OsAFB2 Downregulation via OsmiR393 Overexpression Leads to More Tillers, Early Flowering and Less Tolerance to Salt and Drought in Rice. PLoS ONE.

[104] Gao P., Bai X., Yang L., Lv D., Pan X., Li Y., Cai H., Ji W., Chen Q., Zhu Y. (2011). Osa-MIR393: A Salinity- and Alkaline Stress-Related MicroRNA Gene. Mol. Biol. Rep..

[105] Feng X.-M., You C.-X., Qiao Y., Mao K., Hao Y.-J. (2010). Ectopic Overexpression of Arabidopsis AtmiR393a Gene Changes Auxin Sensitivity and Enhances Salt Resistance in Tobacco. Acta Physiol. Plant..

[106] Porter J.R., Gawith M. (1999). Temperatures and the Growth and Development of Wheat: A Review. Eur. J. Agron..

[107] Sánchez B., Rasmussen A., Porter J. (2013). Temperatures and the Growth and Development of Maize and Rice: A Review. Glob. Chang. Biol..

[108] Jung C., Müller A.E. (2009). Flowering Time Control and Applications in Plant Breeding. Trends Plant Sci..

[109] Wood C., Robertson M., Tanner G., Peacock W., Dennis E., Helliwell C. (2006). The Arabidopsis Thaliana Vernalization Response Requires a Polycomb-like Protein Complex That Also Includes VERNALIZATION INSENSITIVE 3. Proc. Natl. Acad. Sci. USA.

[110] Yan L., Loukoianov A., Blechl A., Tranquilli G., Ramakrishna W., SanMiguel P., Bennetzen J.L., Echenique V., Dubcovsky J. (2004). The Wheat VRN2 Gene Is a Flowering Repressor Down-Regulated by Vernalization. Science.

[111] Mckeown M., Schubert M., Marcussen T., Fjellheim S., Preston J. (2016). Evidence for an Early Origin of Vernalization Responsiveness in Temperate Pooideae Grasses. Plant Physiol..

[112] Fornara F., de Montaigu A., Sánchez-Villarreal A., Takahashi Y., Ver Loren van Themaat E., Huettel B., Davis S.J., Coupland G. (2015). The GI–CDF Module of Arabidopsis Affects Freezing Tolerance and Growth as Well as Flowering. Plant J..

[113] Dhillon T., Pearce S.P., Stockinger E.J., Distelfeld A., Li C., Knox A.K., Vashegyi I., Vágújfalvi A., Galiba G., Dubcovsky J. (2010). Regulation of Freezing Tolerance and Flowering in Temperate Cereals: The VRN-1 Connection. Plant Physiol..

[114] Luan W., Chen H., Fu Y., Si H., Peng W., Song S., Liu W., Hu G., Sun Z., Xie D. (2009). The Effect of the Crosstalk between Photoperiod and Temperature on the Heading-Date in Rice. PLoS ONE.

[115] Anwar M.R., Liu D.L., Macadam I., Kelly G. (2013). Adapting Agriculture to Climate Change: A Review. Theor. Appl. Climatol..

[116] Thines B., Harmon F.G. (2010). Ambient Temperature Response Establishes ELF3 as a Required Component of the Core *Arabidopsis* Circadian Clock. Proc. Natl. Acad. Sci. USA.

[117] Laufs P., Peaucelle A., Morin H., Traas J. (2004). MicroRNA Regulation of the CUC Genes Is Required for Boundary Size Control in Arabidopsis Meristems. Development.

[118] Hendelman A., Stav R., Zemach H., Arazi T. (2013). The Tomato NAC Transcription Factor SlNAM2 Is Involved in Flower-Boundary Morphogenesis. J. Exp. Bot..

[119] Wang J., Bao J., Zhou B., Li M., Li X., Jin J. (2021). The Osa-MiR164 Target OsCUC1 Functions Redundantly with OsCUC3 in Controlling Rice Meristem/Organ Boundary Specification. New Phytol..

[120] Zhang M., Liang S., Hang X., Xiang Y., Cheng Z., Li W., Shi J., Huang L., Sun Y. (2011). Identification of Heavy-Ion Radiation-Induced MicroRNAs in Rice. Adv. Space Res..

[121] Fang Y., Xie K., Xiong L. (2014). Conserved MiR164-Targeted NAC Genes Negatively Regulate Drought Resistance in Rice. J. Exp. Bot..

[122] Li M., Kennedy A., Huybrechts M., Dochy N., Geuten K. (2019). The Effect of Ambient Temperature on Brachypodium Distachyon Development. Front. Plant Sci..

[123] Wang Y., Wang L., Zhou J., Hu S., Chen H., Xiang J., Zhang Y., Zeng Y., Shi Q., Zhu D. (2019). Research Progress on Heat Stress of Rice at Flowering Stage. Rice Sci..

[124] Sharma N., Ruelens P., D’hauw M., Maggen T., Dochy N., Torfs S., Kaufmann K., Rohde A., Geuten K. (2017). A Flowering Locus C Homolog Is a Vernalization-Regulated Repressor in Brachypodium and Is Cold Regulated in Wheat. Plant Physiol..

[125] Hemming M.N., Walford S.A., Fieg S., Dennis E.S., Trevaskis B. (2012). Identification of High-Temperature-Responsive Genes in Cereals. Plant Physiol..

[126] Greenup A.G., Sasani S., Oliver S.N., Talbot M.J., Dennis E.S., Hemming M.N., Trevaskis B. (2010). ODDSOC2 Is a MADS Box Floral Repressor That Is Down-Regulated by Vernalization in Temperate Cereals. Plant Physiol..

[127] Ejaz M., von Korff M. (2017). The Genetic Control of Reproductive Development under High Ambient Temperature. Plant Physiol..

[128] Yong W., Xu Y., Xu W., Wang X., Li N., Wu J., Liang T., Chong K., Xu Z., Tan K. (2003). Vernalization-Induced Flowering in Wheat Is Mediated by a Lectin-like Gene VER2. Planta.

[129] Jagadish S.V.K. (2020). Heat Stress during Flowering in Cereals—Effects and Adaptation Strategies. New Phytol..

[130] Hirabayashi H., Sasaki K., Kambe T., Gannaban R.B., Miras M.A., Mendioro M.S., Simon E.V., Lumanglas P.D., Fujita D., Takemoto-Kuno Y. (2015). QEMF3, a Novel QTL for the Early-Morning Flowering Trait from Wild Rice, Oryza Officinalis, to Mitigate Heat Stress Damage at Flowering in Rice, O. Sativa. J. Exp. Bot..

[131] Corwin D.L. (2021). Climate Change Impacts on Soil Salinity in Agricultural Areas. Eur. J. Soil Sci..

[132] Munns R., Tester M. (2008). Mechanisms of Salinity Tolerance. Annu. Rev. Plant Biol..

[133] Kholodova V., Volkov K., Kuznetsov V. (2010). Plants Under Heavy Metal Stress in Saline Environments. Soil Heavy Metals.

[134] Nikalje G.C., Suprasanna P. (2018). Coping with Metal Toxicity—Cues from Halophytes. Front. Plant Sci..

[135] Lutts S., Kinet J.M., Bouharmont J. (1995). Changes in Plant Response to NaCl during Development of Rice (*Oryza sativa* L.) Varieties Differing in Salinity Resistance. J. Exp. Bot..

[136] Gao J.-P., Chao D.-Y., Lin H.-X. (2007). Understanding Abiotic Stress Tolerance Mechanisms: Recent Studies on Stress Response in Rice. J. Integr. Plant Biol..

[137] Zeng L., Shannon M.C., Grieve C.M. (2002). Evaluation of Salt Tolerance in Rice Genotypes by Multiple Agronomic Parameters. Euphytica.

[138] Hussain S., Zhang J., Zhong C., Zhu L., Cao X., Yu S., Allen Bohr J., Hu J., Jin Q. (2017). Effects of Salt Stress on Rice Growth, Development Characteristics, and the Regulating Ways: A Review. J. Integr. Agric..

[139] Kim W.-Y., Ali Z., Park H.J., Park S.J., Cha J.-Y., Perez-Hormaeche J., Quintero F.J., Shin G., Kim M.R., Qiang Z. (2013). Release of SOS2 Kinase from Sequestration with GIGANTEA Determines Salt Tolerance in Arabidopsis. Nat. Commun..

[140] Wang X., He Y., Wei H., Wang L. (2021). A Clock Regulatory Module Is Required for Salt Tolerance and Control of Heading Date in Rice. Plant Cell Environ..

[141] Wei H., Wang X., He Y., Xu H., Wang L. (2021). Clock Component OsPRR73 Positively Regulates Rice Salt Tolerance by Modulating OsHKT2;1-Mediated Sodium Homeostasis. EMBO J..

[142] Zhang D., Wang Y., Shen J., Yin J., Li D., Gao Y., Xu W., Liang J. (2018). OsRACK1A, Encodes a Circadian Clock-Regulated WD40 Protein, Negatively Affect Salt Tolerance in Rice. Rice.

[143] Gao P., Bai X., Yang L., Lv D., Li Y., Cai H., Ji W., Guo D., Zhu Y. (2010). Over-Expression of Osa-MIR396c Decreases Salt and Alkali Stress Tolerance. Planta.

[144] Zheng G., Wei W., Li Y., Kan L., Wang F., Zhang X., Li F., Liu Z., Kang C. (2019). Conserved and Novel Roles of MiR164-CUC2 Regulatory Module in Specifying Leaf and Floral Organ Morphology in Strawberry. New Phytol..

[145] Ma X., Qiao Z., Chen D., Yang W., Zhou R., Zhang W., Wang M. (2015). CYCLIN-DEPENDENT KINASE G2 Regulates Salinity Stress Response and Salt Mediated Flowering in Arabidopsis Thaliana. Plant Mol. Biol..

[146] Kim S.-G., Kim S.-Y., Park C.-M. (2007). A Membrane-Associated NAC Transcription Factor Regulates Salt-Responsive Flowering via FLOWERING LOCUS T in Arabidopsis. Planta.

[147] Joshi R., Sahoo K.K., Tripathi A.K., Kumar R., Gupta B.K., Pareek A., Singla-Pareek S.L. (2018). Knockdown of an Inflorescence Meristem-Specific Cytokinin Oxidase—OsCKX2 in Rice Reduces Yield Penalty under Salinity Stress Condition. Plant Cell Environ..

[148] Munns R., Rawson H.M. (1999). Effect of Salinity on Salt Accumulation and Reproductive Development in the Apical Meristem of Wheat and Barley. Funct. Plant Biol..

[149] Ghanem M.E., van Elteren J., Albacete A., Quinet M., Martínez-Andújar C., Kinet J.-M., Pérez-Alfocea F., Lutts S. (2009). Impact of Salinity on Early Reproductive Physiology of Tomato (Solanum Lycopersicum) in Relation to a Heterogeneous Distribution of Toxic Ions in Flower Organs. Funct. Plant Biol..

[150] Taoka K.I., Ohki I., Tsuji H., Furuita K., Hayashi K., Yanase T., Yamaguchi M., Nakashima C., Purwestri Y.A., Tamaki S. (2011). 14-3-3 Proteins Act as Intracellular Receptors for Rice Hd3a Florigen. Nature.

[151] Banerjee A., Roychoudhury A. (2017). Abscisic-Acid-Dependent Basic Leucine Zipper (BZIP) Transcription Factors in Plant Abiotic Stress. Protoplasma.

[152] Lu G., Gao C., Zheng X., Han B. (2009). Identification of OsbZIP72 as a Positive Regulator of ABA Response and Drought Tolerance in Rice. Planta.

[153] Tang N., Zhang H., Li X., Xiao J., Xiong L. (2012). Constitutive Activation of Transcription Factor OsbZIP46 Improves Drought Tolerance in Rice. Plant Physiol..

[154] Song S., Wang G., Wu H., Fan X., Liang L., Zhao H., Li S., Hu Y., Liu H., Ayaad M. (2020). OsMFT2 Is Involved in the Regulation of ABA Signaling-mediated Seed Germination through Interacting with OsbZIP23/66/72 in Rice. Plant J..

[155] Shu K., Chen F., Zhou W., Luo X., Dai Y., Shuai H., Yang W. (2018). ABI4 Regulates the Floral Transition Independently of ABI5 and ABI3. Mol. Biol. Rep..

[156] Matamoros M.A., Becana M. (2021). Molecular responses of legumes to abiotic stress: Post-translational modifications of proteins and redox signaling. J. Exp. Bot..

[157] Hashiguchi A., Komatsu S. (2016). Impact of Post-Translational Modifications of Crop Proteins under Abiotic Stress. Proteomes.

[158] Friml J., Gallei M., Gelová Z., Johnson A., Mazur E., Monzer A., Rodriguez L., Roosjen M., Verstraeten I., Živanović B.D. (2022). ABP1–TMK Auxin Perception for Global Phosphorylation and Auxin Canalization. Nature.

[159] Lyzenga W.J., Stone S.L. (2012). Abiotic Stress Tolerance Mediated by Protein Ubiquitination. J. Exp. Bot..

[160] Melo F.V., Oliveira M.M., Saibo N.J.M., Lourenço T.F. (2021). Modulation of Abiotic Stress Responses in Rice by E3-Ubiquitin Ligases: A Promising Way to Develop Stress-Tolerant Crops. Front. Plant Sci..

[161] Kim J.H., Lim S.D., Jang C.S. (2020). Oryza Sativa Drought-, Heat-, and Salt-Induced RING Finger Protein 1 (OsDHSRP1) Negatively Regulates Abiotic Stress-Responsive Gene Expression. Plant Mol. Biol..

[162] Zörb C., Schmitt S., Mühling K.H. (2010). Proteomic Changes in Maize Roots after Short-Term Adjustment to Saline Growth Conditions. Proteomics.

[163] Wei K., Pan S. (2014). Maize Protein Phosphatase Gene Family: Identification and Molecular Characterization. BMC Genom..

[164] Zhang M., Lv D., Ge P., Bian Y., Chen G., Zhu G., Li X., Yan Y. (2014). Phosphoproteome Analysis Reveals New Drought Response and Defense Mechanisms of Seedling Leaves in Bread Wheat (*Triticum aestivum* L.). J. Proteom..

[165] Lv D., Zhu G., Zhu D., Bian Y.-W., Liang X.-N., Cheng Z.-W., Deng X., Yan Y.-M. (2016). Proteomic and Phosphoproteomic Analysis Reveals the Response and Defense Mechanism in Leaves of Diploid Wheat T. Monococcum under Salt Stress and Recovery. J. Proteom..

[166] Zhou L., Liu Y., Liu Z., Kong D., Duan M., Luo L. (2010). Genome-Wide Identification and Analysis of Drought-Responsive MicroRNAs in Oryza Sativa. J. Exp. Bot..

[167] Shan T., Fu R., Xie Y., Chen Q., Wang Y., Li Z., Song X., Li P., Wang B. (2020). Regulatory Mechanism of Maize (*Zea mays* L.) MiR164 in Salt Stress Response. Russ. J. Genet..

[168] An Y., Guo Y., Liu C., An H. (2015). BdVIL4 Regulates Flowering Time and Branching through Repressing MiR156 in Ambient Temperature Dependent Way in Brachypodium Distachyon. Plant Physiol. Biochem..

